# Organelle interplay in host responses and mycobacterial resilience

**DOI:** 10.1038/s44319-026-00836-y

**Published:** 2026-06-18

**Authors:** Shriya Singh, Valentin Berdal, Chloé Deflandre, Zhuoyan Chen, Laurent Kremer, Wassim Daher

**Affiliations:** 1 Centre National de la Recherche Scientifique UMR 9004, Institut de Recherche en Infectiologie de Montpellier (IRIM), Université de Montpellier, Montpellier, France; 2 INSERM, IRIM, Montpellier, France

**Keywords:** Membranes & Trafficking, Microbiology, Virology & Host Pathogen Interaction, Organelles

## Abstract

Mycobacteria exploit host organelles to survive and proliferate within the intracellular environment. In parallel, host cells rely on these organelles to ensure essential biological functions and mount effective immune defenses against invading pathogens. The dynamic competition between host and mycobacteria for control of these organelles represents a central battleground in infection biology and is of increasing scientific interest. Important organelles such as mitochondria, the endoplasmic reticulum, and the Golgi apparatus play pivotal roles in determining the outcome of infection and can tip the balance between pathogen clearance and intracellular survival. Beyond their classical functions in energy production, calcium homeostasis, and protein trafficking, these structures actively participate in immune signaling, metabolism reprogramming, and inflammatory responses. Consequently, they function as powerful defenders against pathogens and, under certain conditions, represent unintentional allies. This review categorizes organelle contributions into two major areas: host-driven cellular defense mechanisms and pathogen-mediated subversion strategies. Recent work in the field is discussed, providing new insights into host-pathogen dynamics and identifying potential therapeutic targets for improved control of mycobacterial infections.

## Abstract (web summary)

Mycobacteria exploit host organelles to survive and proliferate within the intracellular environment. This review discusses host-driven cellular defence mechanisms and pathogen-mediated subversion strategies involving key organelles, also providing insights into potential targets for improved control of mycobacterial infections.

## Graphical abstract

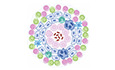

## Introduction

**Figure 1:**
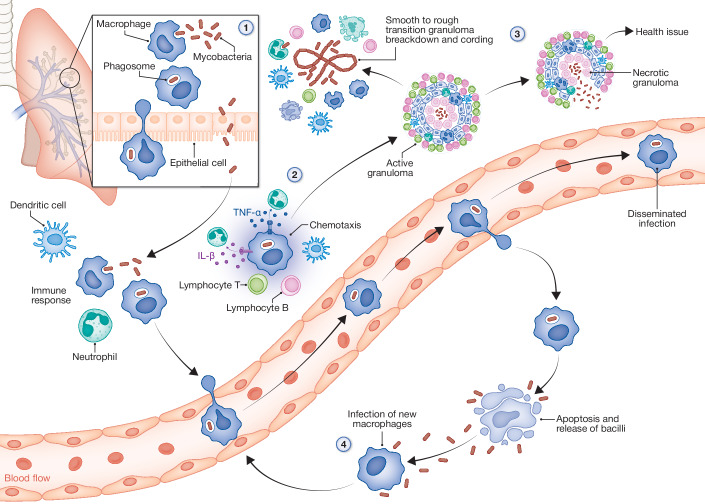
Mycobacterial infection process within the lung microenvironment. This schematic illustrates the spatial and temporal sequence of events through which mycobacteria establish infection in the host lung. (1) Infection is initiated at the alveolar epithelial barrier, where inhaled bacilli interact with innate immune cells, primarily alveolar macrophages. Following phagocytosis, mycobacteria can survive intracellularly within macrophages and traverse the epithelial barrier into the underlying lung tissue. Within the pulmonary parenchyma, infected macrophages release chemokines and cytokines that recruit additional innate and adaptive immune cells (2), promoting the formation of a localized granuloma (3). Granulomas contribute to the containment of the infection but also provide a protected niche that supports long-term bacterial persistence. Pathogenic mycobacteria, particularly rough (R) morphotypes, may additionally form serpentine cords that hinder phagocytosis, enhance extracellular survival, and contribute to tissue damage. In some cases, bacilli escape local containment and disseminate through the bloodstream (4), reaching distant tissues where secondary granulomas can form following release of bacilli during infected cell death or apoptosis.

Mycobacterial infections are widespread worldwide and include both *Mycobacterium tuberculosis* (*Mtb*), the causative agent of tuberculosis (TB), and a diverse group of non-tuberculous mycobacteria (NTM), which differ in their epidemiology, host immune responses, tissue tropism, pathological manifestations, and clinical outcomes (Johansen et al, 2020). TB remains a major global health threat and continues to rank among the top ten causes of death worldwide. According to the Global Tuberculosis Report 2025 published by the World Health Organization, an estimated 10.7 million people (95% UI: 9.9–11.5 million) developed TB and 1.23 million (95% UI: 1.13–1.33 million) died from the disease globally in 2024, making TB the leading cause of death from a single infectious agent. Despite antibiotics and vaccination campaigns, multidrug- and extensively drug-resistant TB remains difficult to treat. In contrast to TB, rapidly and slowly growing NTMs, including *Mycobacterium abscessus*, *Mycobacterium avium* complex, *Mycobacterium kansasii*, and *Mycobacterium marinum*, are increasingly associated with pulmonary, cutaneous, or disseminated infections, particularly in immunocompromised individuals and patients with cystic fibrosis and other comorbidities (Johansen et al, 2020). During infection, mycobacteria can induce granuloma formation, a hallmark of host-pathogen interaction that both contains bacterial growth and provides a niche for persistence. Mycobacteria exhibit remarkable phenotypic plasticity that influences these infection outcomes. For example, in species such as *M. abscessus*, the rough (R) morphotype, unlike the smooth (S) variant, forms serpentine cords that hinder macrophage phagocytosis, promoting extracellular persistence and virulence (Fig. 1) (Bernut et al, 2014). These traits, together with broad intrinsic antibiotic resistance and complex host-pathogen interactions, support chronic persistence and immune evasion (Ghoshal et al, 2024). Concurrently, NTM infections, particularly those caused by *M. abscessus* and *M. avium*, are rising worldwide and more frequently lead to severe disease, especially in immunocompromised individuals (e.g., those with AIDS/HIV or under immunosuppressive therapy) or in patients with comorbidities such as diabetes or malnutrition (Rath et al, 2025).

Upon entry into the lungs *via* aerosolized droplets, mycobacteria are engulfed mainly by alveolar macrophages, yet their intracellular fate varies across species. For example, *Mtb* employs the type VII secretion system ESX-1, whose substrate ESAT-6 can mediate contact-dependent disruption of host membranes, allowing *Mtb* to access the cytosol and evade lysosomal degradation (Conrad et al, 2017; Chandra et al, 2022). In contrast, *M. avium*, which lacks ESX-1, largely remains confined to phagosomes, which can mature to a late endosomal/phagolysosomal stage, initiating inflammatory signaling through TLR7/8-MyD88 and NF-κB pathways (Gidon et al, 2017). Additional virulence factors, including the serine/threonine kinase PknG, the phosphatase SapM, and cell wall components such as lipoarabinomannan (LAM), further modulate phagosomal trafficking and host responses, contributing to pathogen persistence (Warner et al, 2025).

The host response involves recruitment of innate and adaptive immune cells to form granulomas, arising from a combination of defective pathogen clearance in permissive cell types (e.g., alveolar macrophages and neutrophils), local inflammation, and host cell death, which together drive the organized assembly of macrophages, foamy macrophages, T and B cells, dendritic cells (DCs), and fibroblasts (Chandra et al, 2022; Warner et al, 2025). Fibroblasts, although not classical immune cells, contribute structurally by producing extracellular matrix components that stabilize the granuloma and support its persistence, promoting chronic infection. These cellular structures contain the infection while simultaneously providing niches that allow long-term bacterial survival (Fig. 1).

Effective host immunity relies on tightly regulated intracellular networks that detect pathogens while preserving cellular homeostasis. Central to this response are the endo-lysosomal network and the cytosol, which serve as primary compartments for pathogen sensing and the assembly of innate signaling complexes (Nakamura et al, 2014; Miyake et al, 2019). Pathogen recognition occurs when pattern recognition receptors (PRR) such as endosomal Toll-Like Receptors (TLRs) and cytosolic Nucleotide-binding Oligomerization Domain (NOD)-Like Receptors (NLRs) detect microbial components. This happens through docking and assembly of signaling molecules on internal membranes, including those of the endoplasmic reticulum (ER), mitochondria, and Golgi apparatus (Miyake et al, 2019). These organelles play central roles in regulating metabolism, stress responses, cytokine production, and infected-cell fate decisions (Patrick and Watson, 2021). Among them, mitochondria control adenosine triphosphate (ATP) production, reactive oxygen species (ROS) generation, apoptosis, and inflammasome activation; the ER governs protein folding, calcium signaling, and stress responses; and the Golgi apparatus ensures post-translational modifications and targeted secretion of immune mediators. During mycobacterial infection, these organelles, together with endo-lysosomal compartments, are perturbed, resulting in altered host cell physiology, reduced immune detection, and sustained intracellular bacterial persistence (Mohareer et al, 2020; Hüsler et al, 2023).

Importantly, these compartments function as an integrated network rather than isolated entities, whereby inter-organelle communication allows mitochondrial ROS to influence ER stress and calcium-dependent apoptosis, or where lysosomal or Golgi dysfunction impacts immune signaling and trafficking. Mycobacterial infection disturbs this network, causing reduced bactericidal activity while maintaining host cell viability. This leads to metabolic changes and intracellular survival of mycobacteria (Patrick and Watson, 2021; de Souza and Cavalcante, 2022). Infected macrophages often undergo a Warburg-like metabolic shift characterized by increased glycolysis, reduced oxidative phosphorylation (OXPHOS), diminished mitochondrial ROS production, and lipid droplet accumulation, all of which favor bacterial growth and persistence (Hüsler et al, 2023).

**Figure 2:**
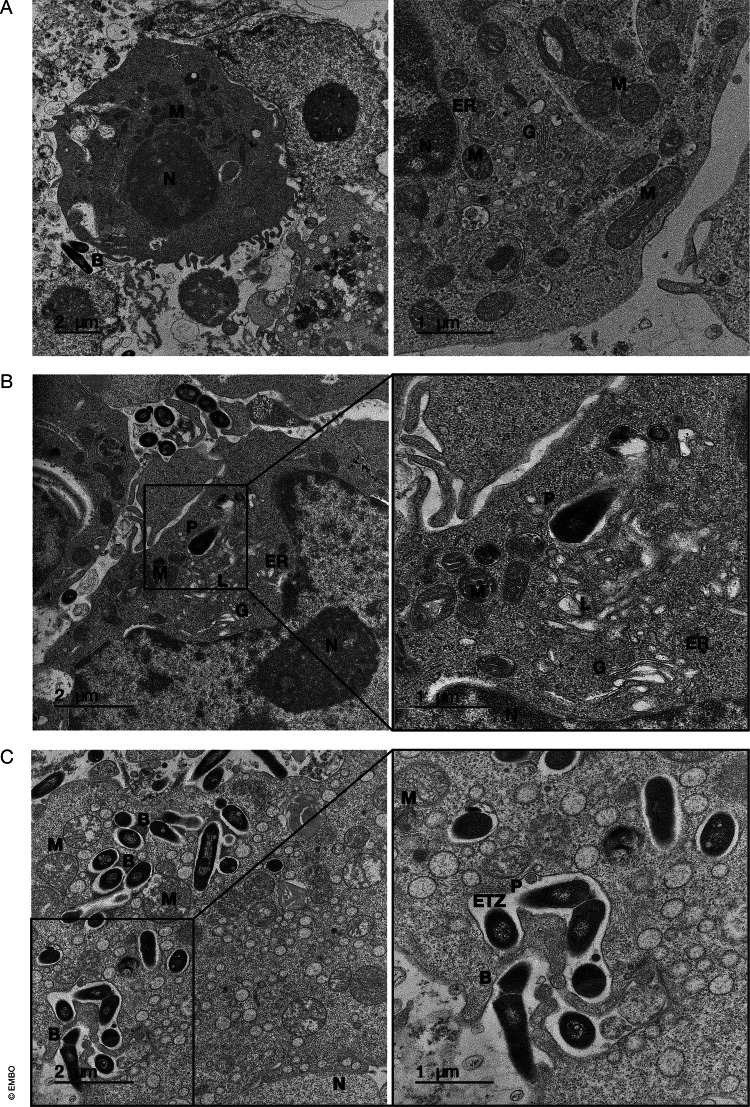
Ultrastructural analysis of *M. abscessus* infection in THP-1 cells. Transmission electron microscopy (TEM) analysis of THP-1 cells differentiated with PMA and infected with *M. abscessus* smooth morphotype (S form) at a multiplicity of infection of 10:1. (**A**) Uninfected THP-1 cell showing a few extracellular bacilli. (**B**) At 4 h post-infection, *M. abscessus* S bacilli are enclosed within phagosomes characterized by tightly apposed phagosomal membranes and close association with mitochondria. (**C**) At 24 h post-infection, phagosomal membranes occasionally display irregularities or partial breaks, suggesting potential phagosome-cytosol communication. Nevertheless, most bacilli remain enclosed within phagosomal compartments, and some exhibit signs of intracellular division. An electron-translucent zone (ETZ), characteristic of the smooth morphotype and associated with surface glycopeptidolipids (GPLs), is observed surrounding the bacilli, consistent with previous observations (Roux et al, 2016). For TEM analysis, cells were fixed overnight at 4 °C in 0.1 M PHEM buffer (pH 7.2) containing 2.5% glutaraldehyde, rinsed in PHEM buffer, and post-fixed for 2 h at room temperature in the dark with 0.5% osmium tetroxide and 0.8% potassium ferrocyanide in PHEM buffer. Samples were dehydrated through graded ethanol solutions (30-100%) followed by absolute acetone using a Leica AMW microwave-assisted tissue processor. Cells were progressively infiltrated with acetone-resin mixtures and embedded in epoxy resin (EMBed 812), which was polymerized at 60 °C. Ultrathin sections (~70 nm) were collected on copper grids using a Leica-Reichert Ultracut E ultramicrotome, contrasted with 1.5% uranyl acetate in 70% ethanol and lead citrate (Reynolds), and examined using a Tecnai F20 transmission electron microscope operated at 120 kV and equipped with a Veleta digital camera (Montpellier RIO Imaging, Montpellier Institute of Neurosciences, INSERM U1298, University of Montpellier, Montpellier, France). These images represent original unpublished data generated in our laboratory. B bacteria, ETZ electron-translucent zone, G Golgi apparatus, N nucleus, M mitochondria, ER endoplasmic reticulum, P phagosome, L lysosome. Scale bars: 2 µm (right panels) and 1 µm (left panels).

Despite intensive research, the molecular mechanisms governing organelle–pathogen crosstalk remain incompletely understood. Upon infection of macrophages with mycobacteria, the phagosome becomes a central hub for intracellular reorganization: mitochondria align around the phagosome to regulate energy production and ROS critical for antimicrobial responses, while the ER and Golgi apparatus position nearby to facilitate membrane trafficking and the secretion of pro-inflammatory cytokines (Fig. 2A–C). In this review, we describe the roles of these organelles during mycobacterial infection and discuss emerging therapeutic strategies aimed at modulating their function to limit bacterial survival and enhance host-directed infection control against TB and NTM diseases.

## Mitochondria in host defense and their subversion by mycobacteria

Mitochondria are active energy factories that produce ATP, support intermediate metabolism, and regulate calcium homeostasis and apoptosis, while also sensing infection and coordinating the host’s antimicrobial signaling. Evidence suggests that mitochondria play a central role in cellular defense during chronic mycobacterial infection. Through ROS generation, OXPHOS, mitochondrial DNA (mtDNA) signaling, apoptosis, inter-organelle networking, and metabolic reprogramming, they convert metabolic energy into an immunological hub. Understanding these multifaceted functions could unveil new avenues for host-directed therapy (HDT) that exploit mitochondrial resilience to enhance immunity against mycobacterial pathogens (Ellzey et al, 2023; Patrick and Watson, 2021).

### Oxidative and nitrosative stress in mycobacterial infection

**Figure 3:**
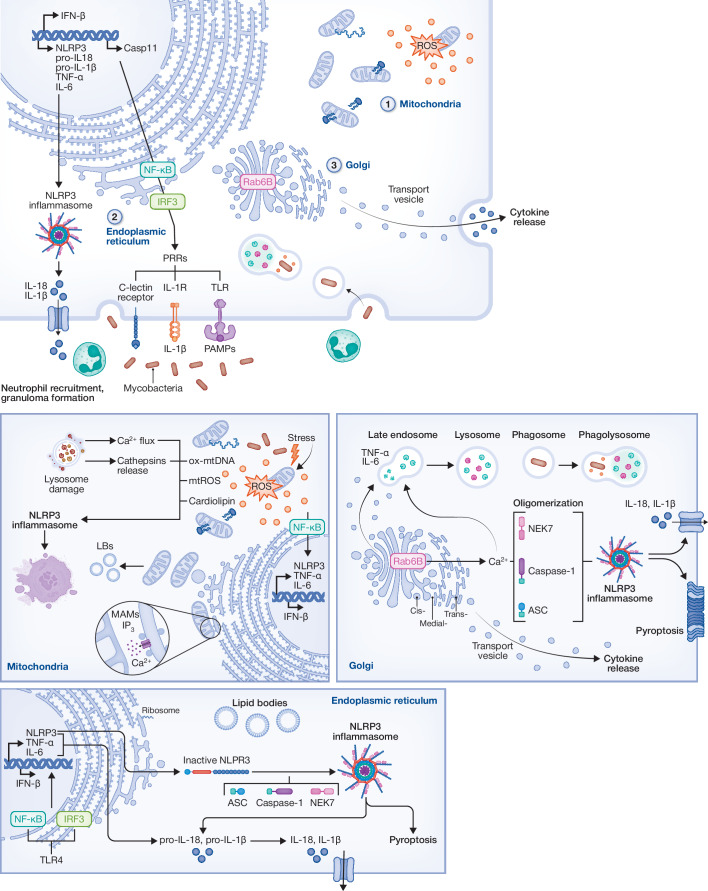
Organelle-specific pathways coordinating host responses to mycobacterial infection. This schematic illustrates how major host organelles orchestrate a multi-layered defense against mycobacteria. (1) Mitochondrial pathway: Mycobacterial infection induces mitochondrial stress, leading to ROS production, release of oxidized mitochondrial DNA (ox-mtDNA), and exposure of cardiolipin, which acts as a danger signal promoting NLRP3 inflammasome priming and activation. MAMs facilitate calcium flux, further enhancing inflammasome signaling and apoptosis, while lysosomal damage and cathepsin release from disrupted late endosomes and lysosomes converge with these signals. NF-κB activation downstream of pathogen recognition receptors primes the inflammasome by upregulating NLRP3, pro-IL-1β, pro-IL-18, TNF-α, IL-6, and IFN-β. LBs, are also regulated by the mitochondria and the endoplasmic reticulum, playing a role in lipid storage and cellular stress responses. (2) ER/NLRP3 inflammasome pathway: In resting cells, NLRP3 resides in an inactive state at the ER; upon activation, it recruits ASC (Apoptosis-associated speck-like protein containing a CARD, i.e., caspase activation and recruitment domains) and NEK7 (NIMA-related kinase 7) to assemble the inflammasome and activate caspase-1, which cleaves pro-IL-1β and pro-IL-18 into mature IL-1β and IL-18, leading to pyroptosis, neutrophil recruitment, and granuloma formation. (3) Golgi pathway: Via glycosylation processes, the Golgi apparatus regulates cytokine processing and vesicular trafficking, with Rab6B and calcium-dependent transport directing TNF-α and IL-6 from the Golgi to late endosomes, lysosomes, phagosomes, and phagolysosomes, supporting both cytokine secretion and phagosome maturation, thereby promoting autophagy and intracellular bacterial clearance. Collectively, these organelle-mediated pathways integrate danger signals, inflammasome activation, cytokine secretion, and phagosome maturation to mount a coordinated antimycobacterial response. NLRP3 NOD-like receptor family, pyrin domain containing 3, IRF3 interferon regulatory factor 3, Casp11 caspase-11, NF-κB nuclear factor kappa-light-chain-enhancer of activated B cells, IP₃ inositol 1,4,5-trisphosphate, IFNAR interferon-α/β receptor.

Upon infection with mycobacteria, macrophage-mediated innate immunity rapidly initiates antimicrobial signaling cascades and mitochondrial metabolism is swiftly upregulated, leading to the production of ROS (mtROS) (Fig. 3). Generated largely through reverse electron transport at complex I, mtROS create a bactericidal intracellular environment that promotes pathogen clearance. Beyond their direct antimicrobial activity, mtROS function as second messengers, activating transcription factors like NF-κB and inducing the production of pro-inflammatory cytokines, including TNF (Roca and Ramakrishnan, 2013; Singhal et al, 2014) (Fig. 3). TNF-driven mtROS can initially enhance microbicidal activity but may also trigger necroptosis, releasing bacteria into the extracellular environment, thus contributing to mycobacterial pathogenesis (Roca and Ramakrishnan, 2013). Antioxidant systems, including superoxide dismutase-2 (SOD2) and glutathione, together with mitochondrial regulators, such as SIRT3 and IDH2, finely tune mtROS levels to maximize bacterial killing while limiting oxidative damage and necrotic cell death (Smulan et al, 2021; Yabaji et al, 2020; Singhal et al, 2014).

ROS generation and signaling are further influenced by the availability of transition metals in the phagosome. Iron, copper, and zinc serve as essential cofactors for enzymatic and structural functions in both host and pathogen, but can generate ROS when present in excess (Marcela Rodriguez and Neyrolles, 2014; Cornelis et al, 2011). During infection, host macrophages modulate metal availability through nutritional immunity and metal toxicity, increasing copper and zinc concentrations while limiting iron and manganese to restrict bacterial growth (Neyrolles et al, 2015; Healy et al, 2021). These changes in metal availability influence ROS homeostasis: for example, iron depletion can enhance ROS generation *via* the Fenton reaction, amplifying oxidative stress and bactericidal effects (Li et al, 2022; Capdevila et al, 2024). *Mtb* responds through metal uptake, storage, and efflux systems, as well as redox-sensitive regulators such as WhiB3, which modulate responses to ROS and reactive nitrogen species (RNS) (Mehta and Singh, 2019). These observations highlight that mitochondrial ROS and phagosomal metal flux intersect to shape oxidative stress during infection, reinforcing host antimicrobial defenses without implying purposeful action by the bacterium. In parallel, nitric oxide (NO), produced by the inducible nitric oxide synthase (iNOS) in macrophages and other immune cells, is a key mediator of host defense (Yang et al, 2009; Chan et al, 2001). In mice, NO contributes directly to bacterial killing, and iNOS deficiency increases bacterial dissemination and mortality; in humans, NO also shows antimycobacterial effects. Beyond direct killing, NO activates HIF-1α and modulates NF-κB to promote antimicrobial programs while limiting inflammation (Braverman and Stanley, 2017). It restrains immunopathology by inhibiting NLRP3 inflammasome activation and suppressing neutrophil-driven inflammation, which can otherwise create permissive niches for *Mtb* replication (Mishra et al, 2013, 2017). Macrophage-mediated inflammatory responses enhance NO production in surrounding cells, reducing bacterial survival and supporting control of latent infection (Yang et al, 2016).

Mitochondrial stress can also lead to the release of mtDNA into the cytosol, where it is detected by cyclic guanosine monophosphate (GMP) – adenosine monophosphate (AMP) synthase (cGAS). Activation of the cGAS-STING-TBK1-IRF3 axis triggers type I interferon responses, linking mitochondrial metabolism to innate immune activation, autophagy, and macrophage polarization (Watson et al, 2015; Ding et al, 2024; Ruangkiattikul et al, 2017). Virulent *Mtb* can limit mtDNA release and modulate mitochondrial signaling hubs using CpsA, a virulence factor that alters cytosolic DNA levels and TBK1/IRF3 phosphorylation, leading to changes in IFN-I production (Ding et al, 2024). Notably, the functional outcome of cGAS-STING activation is context-dependent: while it promotes antibacterial activity in some macrophage and NTM models (*M. avium* complex) (Ruangkiattikul et al, 2017), it is dispensable for host protection in vivo during *Mtb* infection, thus underpinning the complexity of this pathway (Marinho et al, 2018). Beyond their role in inflammatory signaling, mitochondria also serve as central platforms for initiating host cell death programs that restrict intracellular mycobacterial survival.

### Apoptosis as a containment strategy

Apoptosis is a major host mechanism for limiting intracellular pathogen survival. In macrophages infected with *Mtb*, the intrinsic apoptotic pathway is driven by mitochondrial outer membrane permeabilization (MOMP), a key event that results in the release of cytochrome c and other pro-apoptotic factors from the mitochondrial intermembrane space (Green and Kroemer, 2004). Activation of the BH3-only protein BID leads to the engagement of the pro-apoptotic Bcl-2 family members BAX and BAK, which oligomerize at the outer mitochondrial membrane and induce MOMP (major outer membrane porin) (Chipuk and Green, 2008). This process promotes cytochrome c release, activation of caspase-9, and subsequent activation of executioner caspases, ultimately leading to programmed cell death of infected cells (Bossy-Wetzel et al, 1998). In *Mtb* infection, particularly with attenuated strains, apoptosis is associated with BAX-dependent MOMP without induction of mitochondrial permeability transition (Chen et al, 2006). Clearance of dying cells by DCs further amplifies host defense by promoting antigen presentation and T-cell differentiation. Thus, tightly regulated apoptosis removes intracellular niches that sustain bacterial replication while reinforcing adaptive immune responses. In contrast, the collapse of mitochondrial membrane potential and severe ATP depletion favor necrotic cell death, giving rise to the uncontrolled release of viable bacilli into surrounding tissues. The balance between apoptosis and necrosis is strongly influenced by mitochondrial metabolism: intact OXPHOS favors apoptotic programs, whereas a metabolic shift toward glycolysis predisposes cells to necrotic death (Pagán et al, 2022). Coordinated mitochondrial dynamics, *via* fission–fusion integrity, are essential in determining cell fate and infection outcome (Mohareer et al, 2020). Fusion proteins MFN1, MFN2, and OPA1 regulate mitochondrial elongation, while DRP1 drives fission. Upon pathogen encounter, macrophage mitochondria elongate, enhancing respiratory capacity and mtROS production to support antimicrobial signaling (Ning et al, 2021). Excessive fission, however, disrupts mitochondrial integrity, reduces OXPHOS efficiency, and impairs immune function. Consistently, MFN2 activity promotes mtROS production and enhances macrophage bactericidal functions, leading to restriction of mycobacterial growth. This underscores the direct link between mitochondrial morphology, pro-inflammatory activation, and host immune competence (Tur et al, 2020). In this context, mitochondrial quality-control mechanisms are essential for maintaining a functional organelle network that favors apoptosis over necrosis and supports effective antimicrobial immunity.

### Metabolic reprogramming toward host protection

**Figure 4:**
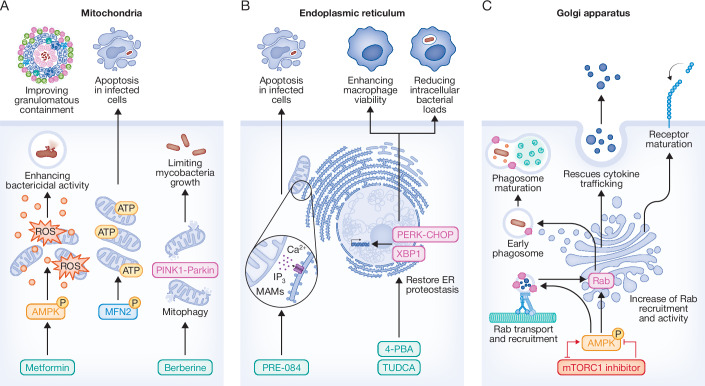
Organelle-targeted therapeutic strategies against disseminated mycobacterial infection. (**A**) Mitochondrial targeting: Metformin activates AMPK, inducing ROS production to enhance antibacterial activity and maintain granuloma integrity. Activated MFN2 promotes ATP production and apoptosis in infected cells. Berberine stimulates the PINK1-Parkin pathway, promoting mitophagy and limiting bacterial growth. (**B**) ER–mitochondria modulation: PRE-084 restores calcium flux at MAMs, reactivates apoptosis, and improves oxidative metabolism and immune responsiveness. TUDCA and 4-PBA restore ER proteostasis, enhancing macrophage viability and reducing intracellular bacterial loads. (**C**) Golgi and phagosome maturation pathways: AMPK activation or mTORC1 inhibition regulates Rab GTPase cycling, promoting phagosome maturation, rescuing cytokine trafficking, and restoring receptor maturation. Rab GTPases originating from the Golgi act as molecular switches that indirectly regulate phagosome maturation by recruiting downstream effector proteins rather than interacting directly with the phagosome. Following bacterial uptake, early phagosomes acquire Rab5, which coordinates early endosomal fusion events. During maturation, Rab conversion from Rab5 to Rab7 occurs, and Golgi-derived vesicles supply membrane and lysosomal components to the developing phagosome. The apparent Rab-phagosome connection reflects this indirect pathway, whereby Rab proteins recruit tethering complexes such as CORVET (early stages) and HOPS (late stages), which bridge phagosomes with incoming endosomal and Golgi-derived vesicles. These tethering complexes subsequently enable SNARE-mediated membrane fusion, allowing delivery of degradative enzymes and acidifying components. Thus, tethering machinery and associated effectors act as essential intermediates between Rab GTPases and the phagosome, driving stepwise maturation. AMPK AMP-activated protein kinase.

Mitophagy is essential for maintaining a functional mitochondrial network capable of sustaining effective immune responses. This process is primarily initiated through the PINK1-Parkin pathway, in which depolarized organelles are marked for degradation, while alternative pathways involving BNIP3 and NIX operate under hypoxic conditions (Liu et al, 2024). During *Mtb* infection, timely mitophagy prevents excessive ROS release and necrotic cell death, preserving energy homeostasis and limiting pathogen survival. Beyond the elimination of dysfunctional organelles, mitochondrial fitness relies on close communication with other cellular compartments. In particular, mitochondria-associated membranes (MAMs) (Fig. 3) linking mitochondria and the ER facilitate calcium exchange critical for inflammasome activation and apoptosis (Bui et al, 2026). Excess calcium release through IP3 receptors (Figs. 3 and 4) triggers cytochrome c release, coupling energy metabolism to immune defense, whereas disruption of MAMs integrity weakens host protection and favors bacterial survival. Mitochondrial signaling also intersects with autophagy pathways to clear pathogens: mtROS activates AMPK, leading to mTORC1 inhibition and induction of autophagy (Pagán et al, 2022) (Fig. 4). Sequestration of *Mtb* within LC3-positive vesicles restricts bacterial survival, while mitophagy prevents the accumulation of damaged mitochondria that could otherwise release DAMPs (damage-associated molecular patterns). Together, these quality-control mechanisms balance energy metabolism, inflammation, and pathogen clearance, highlighting mitochondria as central regulators of host-protective metabolic reprogramming (Croock et al, 2025; Priyanka et al, 2024; Yadav et al, 2025).

In parallel, host lipid metabolism undergoes profound reprogramming during mycobacterial infection, associated with the formation of lipid bodies (LBs) within macrophages (Fig. 3). These organelles act as reservoirs of triacylglycerols (TAGs) and cholesterol esters, supplying energy and metabolic intermediates that sustain both host immune function and bacterial survival. LBs accumulate in foamy macrophages characteristic of TB granulomas and are induced by immune and metabolic signals during infection in vitro and in vivo, where neutral lipids form the storehouse of these dynamic organelles. LBs are closely associated with mitochondria and ER membranes, positioning them at the nexus of cellular stress responses: mitochondrial ROS and calcium flux regulate LB biogenesis, while ER stress and unfolded protein response (UPR) signaling influence lipid synthesis and storage. Mycobacteria import fatty acids from host-derived LBs and store them as intrabacterial lipid inclusions (ILIs), which serve as critical energy reserves under nutrient-limiting conditions and are associated with long-term persistence in a dormant-like non-replicating state (Dargham et al, 2023; Mallick et al, 2021). Recent analyses have identified a conserved core of ILI-associated proteins across TAG-producing actinobacteria, highlighting the molecular machinery that governs bacterial lipid storage, mobilization, and metabolic adaptation (Dargham et al, 2023). This LB-ILI axis intersects with autophagic and inflammasome pathways: LBs can sequester lipophilic signaling molecules and modulate AMPK–mTORC1 activity (Fig. 4), thereby influencing mitophagy and phagosome maturation, while ILIs buffer bacterial metabolism against oxidative stress and ER-mediated host defenses. By integrating host lipid storage, organelle stress responses, and bacterial metabolic adaptation, the LB-ILI interplay exemplifies a finely tuned metabolic interface through which mitochondria, ER, and pathogen co-regulate infection outcomes (Mallick et al, 2021).

Collectively, these observations suggest that mitochondrial quality control, inter-organelle crosstalk, autophagy, and lipid metabolism are intertwined processes that orchestrate the metabolic landscape of infected macrophages. The dynamic coordination of these pathways allows host cells to restrict bacterial replication while balancing energy production and inflammatory signaling. However, mycobacterial pathogens interact with these metabolic nodes, causing modifications that are associated with bacterial survival and long-term persistence in the host.

### Inflammasome activation and signaling

Mitochondria coordinate inflammasome activation through multiple mechanisms beyond mtDNA release. Externalized ROS and cardiolipin on damaged mitochondrial membranes provide a platform for NLRP3 inflammasome assembly, promoting the maturation of IL-1β and IL-18 (Chai et al, 2025) (Fig. 3). These cytokines recruit neutrophils to infection sites and support granuloma formation, a hallmark of mycobacterial pathogenesis. Mitochondrial metabolic shifts further shape host defense. During *Mtb* infection, macrophages initially polarize toward a pro-inflammatory M1 phenotype, increasing glycolysis while maintaining mitochondrial OXPHOS and ROS production to support antimicrobial functions (Shi et al, 2016; Gleeson et al, 2016; Tannahill et al, 2013). Accumulation of tricarboxylic acid (TCA) cycle intermediates, such as succinate, stabilizes HIF-1α and enhances IL-1β production, whereas itaconate limits excessive inflammation (Diskin and Pålsson-McDermott, 2018; Mills and O’Neill, 2014). As infection progresses, macrophages shift toward an anti-inflammatory M2 phenotype, characterized by fatty acid oxidation, restored OXPHOS, and LB formation, providing nutrients for bacterial survival (Shi et al, 2019; Zhang et al, 2020). Modulating host mitochondrial metabolism, for example, by enhancing glycolysis or blocking lipid accumulation can, therefore, skew immune responses toward pathogen clearance (Allison et al, 2011). Mitochondria also cooperate with other organelles, including the Golgi apparatus and peroxisomes, to coordinate cytokine secretion (Fig. 3). Mitochondrial ATP fuels vesicular trafficking, while ROS guides redox-dependent cytokine folding (Canton et al, 2021; Tur et al, 2020). Disrupted mitochondrial energy dynamics impair IL-6 and TNF-α secretion, whereas restoring mitochondrial function enhances inter-organelle communication and cytokine delivery. Thus, mitochondria appear as central hubs that integrate metabolism, inflammatory signaling, and cell death to coordinate antimicrobial immunity.

### Mitochondrial subversion by mycobacteria

Mitochondria play central roles in host defense by regulating ROS production, apoptosis, mitophagy, and metabolic signaling. Mycobacteria perturb these functions, inducing metabolic and structural alterations that promote sustained intracellular survival, chronic infection, and reduced immune clearance. Following infection, macrophages undergo metabolic reprogramming that links mitochondrial pyruvate import to complex I-dependent ROS generation *via* reverse electron transport, contributing to control of bacterial burden, while a shift from OXPHOS toward aerobic glycolysis, mediated in part by HIF-1α, leads to LB accumulation, foamy macrophage formation, and environments that support bacterial persistence (Røst et al, 2022; Genoula et al, 2020; Daniel et al, 2011; Peyron et al, 2008). *Mtb* infection also alters mitochondrial dynamics by promoting fission through MFN2 degradation and DRP1 (dynamin-related protein 1) mediated fragmentation, impairing ATP production, ROS generation, calcium buffering, and ER contacts, whereas MFN1-mediated fusion enhances autophagy and restricts bacterial survival (Lee et al, 2019; Ning et al, 2021).

Pathogenic mycobacteria affect host cell death pathways, contributing to the maintenance of the intracellular niche. *Mtb* infection has been shown to inhibit macrophage cell death through multiple virulence factors, including the NADH (reduced nicotinamide adenine dinucleotide) dehydrogenase subunit NuoG, the Ser/Thr protein kinase PknE, and the SecA2 secretion system, while upregulating anti-death proteins BCL-2 and MCL-1 and suppressing caspase-9, resulting in delayed intrinsic apoptosis and prolonged macrophage survival (Lee et al, 2009; Abebe et al, 2011; Wang et al, 2016; Hinchey et al, 2007; Jayakumar et al, 2008; Velmurugan et al, 2007). BNIP3/NIX-mediated mitophagy contributes to the clearance of damaged mitochondria, limits mtROS, and maintains ATP levels, whereas PE/PGRS proteins perturb mitochondrial membranes, ROS production, calcium homeostasis, and apoptotic signaling, which was shown to modulate Bax and Bim pathways in macrophages (Matsumura et al, 2023; Medha et al, 2023). Calcium signaling is further altered by PE_PGRS1, which reduces intracellular calcium levels and attenuates ER stress signaling (Yu et al, 2023). Necroptotic and mTORC1-dependent pathways are also partially modulated, contributing to the preservation of granuloma structure, limited macrophage death, and sustained bacterial persistence (Pagán et al, 2022; Stutz et al, 2018). *Mtb* infection disrupts mitochondrial communication with other organelles, including ER–mitochondria contact sites, impairing calcium flux, NLRP3 inflammasome activation, and ATP-dependent cytokine secretion, collectively dampening innate immune responses (Rastogi and Briken, 2022; Mohareer et al, 2020; Pereira et al, 2022). Interference with antigen presentation via ESX-1/ESAT-6, PDIM, and LINC02528-TOMM22 reduces mitochondrial peptide display on MHC class I, limiting dendritic cell cross-priming and CD8⁺ T-cell activation (Hinchey et al, 2007; Xu et al, 2025; Wiens and Ernst, 2016; Cumming et al, 2018). Redox metabolism is also altered, with changes in NAD⁺/NADH ratios, SIRT1 downregulation, and accumulation of immuno-metabolites such as itaconate, resulting in suppressed ROS production and inflammatory signaling that favor a tolerant macrophage phenotype (Cheng et al, 2017; Zhang et al, 2023; Kim et al, 2022; Peace and O’Neill, 2022). Proteostasis is maintained *via* the ClpP1P2 protease, whose activity is required for mitochondrial quality control and intracellular survival (Illigmann et al, 2021). Collectively, these findings demonstrate that mitochondrial subversion is not an isolated event but a coordinated program affecting antigen presentation, metabolism, inter-organelle signaling, and stress responses.

## Endoplasmic reticulum as the cellular stress and immune hub

The ER coordinates protein folding, calcium homeostasis, and immune signaling, serving as a central hub for cellular defense. During mycobacterial infection, the ER undergoes stress responses that alter host processes, including activation of the unfolded protein response, which can influence cell survival and bacterial persistence, reflecting how host pathways are affected during infection.

### UPR-driven inflammatory responses

The UPR is a cellular stress response triggered by the accumulation of misfolded or unfolded proteins in the ER. It functions to restore ER homeostasis by temporarily reducing protein synthesis, enhancing the production of molecular chaperones, and promoting the degradation of misfolded proteins. Mycobacterial effectors, which are secreted proteins that manipulate host cell processes, can activate the UPR to modulate host immune responses. For example, the *Mtb* CdhM (mycobacterium protein cell adhesion molecule M-cadherin) directly induces ER stress, causing membrane alterations and UPR activation (Xu et al, 2022). Similarly, the ER-resident host protein Herp, a key component of ER-associated degradation (ERAD), is induced during *Mtb* infection *via* ATF6-dependent ER stress. Herp suppresses ROS-mediated autophagy, thereby promoting intracellular survival of mycobacteria, while its depletion enhances ER stress responses and reduces bacterial persistence (Son et al, 2023). Other mycobacterial effectors, such as PE_PGRS1, highlight the interplay between ER stress, calcium homeostasis, and apoptosis. PE_PGRS1, a calcium-binding protein, reduces intracellular calcium levels and dampens the PERK-eIF2α-ATF4 branch of the UPR, thereby limiting macrophage apoptosis and promoting intracellular survival of mycobacteria (Yu et al, 2023). Collectively, these studies indicate that specific mycobacterial effectors interact with the ER and modulate host processes in ways that can influence bacterial survival.

Building on this, mycobacterial effectors modulate UPR plasticity, which can influence macrophage survival and the balance of pro-apoptotic or pro-inflammatory responses. PE/PPE proteins, particularly those of the PE_PGRS subgroup, are central to this modulation (Grover et al, 2018; Long et al, 2019; Xie et al, 2021). For example, Rv0297 (a PE_PGRS protein) localizes to the ER and triggers unfolded protein response-driven, TLR4-dependent apoptosis, a mechanism that may facilitate mycobacterial dissemination rather than effective bacterial clearance (Grover et al, 2018). In contrast, PE_PGRS62 disrupts ER stress-mediated apoptotic signaling in macrophages, reducing apoptosis and thereby promoting intracellular mycobacterial survival while altering host cytokine responses (Long et al, 2019). These observations are consistent with evidence from TB granulomas showing that ER stress markers, including CHOP (C/EBP homologous protein), phosphorylated IRE1α, eIF2α, and ATF3, are enriched in macrophage-rich areas, where they co-localize with apoptotic cells, highlighting the relevance of UPR-driven cell death during *Mtb* infection in vivo (Seimon et al, 2010). This fine-tuned modulation of ER stress can be considered an “alarm-quenching” strategy, enabling *Mtb* to persist within macrophages by suppressing apoptosis and dampening pro-inflammatory host signaling.

### Calcium-mediated immune cell activation

Mycobacterial effectors, such as the CtpF calcium-transporting ATPase, perturb host calcium homeostasis, suppress mTOR-dependent autophagy, and thereby enhance *Mtb* survival within macrophages (Garg et al, 2020). Co-chaperones such as BAG2 modulate ER stress during *Mtb* infection, promoting autophagic flux and selective reticulophagy (autophagy targeted to the ER), and thereby protecting macrophages from apoptosis (Liang et al, 2020). ER stress can induce apoptosis during *Mtb* infection, involving pathways such as eIF2α/CHOP and STING-TBK1-IRF3, highlighting its dual role in controlling bacterial growth while potentially facilitating dissemination (Cui et al, 2016; Lim et al, 2011). During co-infections with *Mtb* and HIV-1, calcium signaling interacts with TLR2 pathways in a complex manner: calcium released from intracellular stores inhibits macrophage cell death, whereas calcium influx from the extracellular environment can trigger programmed cell death, resulting in finely tuned regulation of macrophage survival (Mehto et al, 2015) (Fig. 3). *Mtb* infection affects the interplay between calcium signaling and the UPR *via* PE_PGRS proteins, such as Rv0297 and PE_PGRS41, driving modulation of pro-inflammatory and pro-apoptotic signaling, sustained macrophage viability, and conditions associated with long-term bacterial persistence (Sharma et al, 2021; Deng et al, 2017). A recent review highlights that calcium signaling and the UPR intricately regulate autophagy during mycobacterial infection, influencing granuloma formation and the balance between host defense and bacterial survival (Li et al, 2025).

### Antigen loading and peptide processing

The ER is essential for peptide loading and antigen presentation. *Mtb* can interfere with antigen processing pathways in macrophages, including ER-associated machinery such as TAP (transporter associated with antigen processing), tapasin, and ER aminopeptidases (ERAPs), by limiting peptide availability for MHC I loading. This reduces MHC I antigen presentation and can impair CD8⁺ T-cell recognition, contributing to immune evasion during chronic infection (Tobian et al, 2003; Witt, 2023; Saulle et al, 2020) (Fig. 3). Alternative antigen presentation pathways, including MR1 and HLA-E, rely on proper ER-associated machinery such as tapasin, TAP, and retrotranslocation for ligand loading. *Mtb* infection has been associated with altered regulation of these processes, including reduced MR1 and HLA-E surface expression and diminished activation of MAIT cells and HLA-E-restricted CD8⁺ T cells, resulting in decreased immune detection and cytotoxic responses (Kim and Karamooz, 2022). *Mtb* infection has been associated with altered ER trafficking, leading to changes in antigen presentation. Several secreted proteins, such as Mpt64, localize to the ER during infection and disrupt ER functions, including the UPR, which can impair the transport of peptides to MHC class I and II molecules and hinder effective T-cell activation (Stamm et al, 2019; Augenstreich and Briken, 2020). Moreover, proteins such as PE_PGRS41 act as virulence factors that manipulate host cell functions, altering ER-associated processes and reducing MHC complex maturation, which can impair T-cell recognition and promote mycobacterial survival in macrophages (Deng et al, 2017). Alterations in antigen transport, ER stress, and calcium homeostasis during *Mtb* infection contribute to cellular conditions that are associated with bacterial survival and persistence.

## Golgi apparatus: the immune logistic hub and target of mycobacterial subversion

The Golgi apparatus is a central organelle in the secretory pathway, responsible for modifying, sorting, and trafficking proteins and lipids to their appropriate cellular destinations. In macrophages, activation of PRR triggers phagosome formation and transcription of pro-inflammatory cytokines (e.g., TNF-α, IL-6, and IL-1β), while immune stimulation simultaneously enhances intracellular transport machinery, such as Rab GTPases (Maphasa et al, 2020; Pei et al, 2012; Kaminska et al, 2024). Newly synthesized cytokines traffic from the rough ER to the Golgi, where post-translational modifications and sorting direct them to secretory pathways (Fig. 3). In parallel, non-classical routes, including autophagy-related and inflammasome-dependent secretion, also contribute to cytokine release (Kaminska et al, 2024). During *Mtb* infection, alterations in trafficking and autophagy pathways are associated with intracellular persistence, highlighting the Golgi as a key node in host-pathogen interactions (Maphasa et al, 2020).

### Glycosylation to stabilize immune receptors

Golgi-mediated glycosylation is a critical step in stabilizing, maturing, and regulating the function of immune receptors and secreted mediators. Glycosyltransferases and glycosidases act sequentially along the *cis*-, *medial*-, and *trans*-Golgi cisternae to remodel oligosaccharide chains initially added in the ER, a process tightly coordinated with vesicular trafficking to maintain enzyme localization (Frappaolo et al, 2020; Hsieh et al, 2025) (Fig. 3). These modifications influence protein folding, receptor conformation, trafficking efficiency, and resistance to degradation, ultimately determining receptor stability and ligand-binding capacity (Frappaolo et al, 2020).

In macrophages, proper glycosylation ensures accurate trafficking and secretion of cytokines and other glycoproteins, preserving their bioactivity and signaling potential (Trzos et al, 2023). Disruption of Golgi architecture or glycosylation enzyme distribution can lead to defective glycan synthesis, altered receptor maturation, and impaired immune signaling (Fig. 3). Glycosylation and vesicular trafficking are, therefore, tightly interconnected: the movement of enzymes and cargo through Golgi cisternae dictates glycan processing, while retrograde and anterograde transport maintain the machinery required for correct receptor maturation and immune function.

### Trafficking of immune receptors and enzymes

Rab6B plays a pivotal role by controlling vesicle budding, directionality, and fusion for cargos carrying key immune effectors (Domínguez Cadena et al, 2021; Pei et al, 2012). In macrophages, Rab6B localizes to the Golgi complex and facilitates secretion of TNF-α during mycobacterial infection, ensuring correct localization and bioactivity without affecting TNF mRNA or intracellular protein levels (Domínguez Cadena et al, 2021) (Fig. 3). Through these mechanisms, surface receptors are delivered to the plasma membrane to initiate signaling, while enzymes and other effectors are directed to intracellular compartments, including phagosomes, where they support maturation and microbicidal functions. Rab6B thus provides spatial and temporal precision to immune responses, whose effectiveness depends on organelles that function both as defense hubs and as targets for mycobacterial subversion.

### Golgi subversion and vesicle-mediated immune modulation by *Mtb*

Mycobacterial infection is associated with modulation of Golgi-associated Rab GTPases, particularly Rab6B, which regulates post-Golgi trafficking of TNF-α. Rab6B facilitates TNF-α secretion without affecting its mRNA or intracellular protein levels, indicating involvement of the host secretory pathway in regulating pro-inflammatory signaling during infection (Domínguez Cadena et al, 2021; Pei et al, 2012). In parallel, mycobacterial lipoglycans, especially mannose-capped LAM, modulate host immune signaling and dampen inflammatory responses (Nigou et al, 2003). *Mtb* also releases extracellular vesicles containing proteins, lipoproteins, and lipoglycans, which can be exported from infected macrophages and interact with uninfected cells, influencing immune responses at a distance (Layre, 2020; Gupta et al, 2023; Athman et al, 2015; Salgueiro et al, 2024). These mechanisms, interfering with Rab6B-dependent trafficking, modulating host signaling *via* LAM, and exporting bacterial components through vesicles, act together to create a permissive intracellular niche, impair local cytokine responses, and promote bacterial persistence and immune evasion (Chandra et al, 2022; Layre, 2020).

## Inter-organelle crosstalk in host defense and mycobacterial subversion

Mitochondria–ER contact sites (MERCs) and ER–Golgi trafficking constitute central hubs for immune regulation and intracellular pathogen manipulation. MERCs, defined by a 15–50 nm proximity between mitochondria and the ER, function as dynamic signaling platforms coordinating calcium flux, lipid metabolism, apoptosis, autophagosome formation, and inflammasome assembly (Martinvalet, 2018; Giacomello and Pellegrini, 2016; Pereira et al, 2022). They are essential for innate defense, regulating mitochondrial antiviral signaling (MAVS) and serving as assembly sites for the NLRP3 inflammasome, which is activated by mitochondrial ROS. Intracellular pathogens, including *Mtb*, perturb calcium homeostasis, alter metabolic stress signaling, and affect mitochondrial dynamics, which can limit inflammasome activation (Omotade and Roy, 2019). ER–Golgi trafficking, mediated by COPII vesicles and SNARE proteins, is critical for the secretion and maturation of immune mediators, yet is frequently hijacked by intracellular bacteria to establish pathogen-containing vacuoles and persistent niches (Clemente et al, 2023; Chatterjee et al, 2024). *Mtb* affects multiple organelles: it modulates mitochondrial LB metabolism, bringing about increased fatty acid oxidation; induces ER stress and influences the unfolded protein response in ways that can delay apoptosis; and alters vesicular trafficking, which is associated with maintained host cell viability (Menon et al, 2019; Stamm et al, 2019; Sankar et al, 2024; Rankine-Wilson et al, 2021). Secreted effectors such as Mpt64 localize to the ER, disrupting the unfolded protein response and further remodeling the host intracellular environment to favor bacterial survival (Stamm et al, 2019). Collectively, this illustrates how *Mtb* and other intracellular pathogens affect multiple organelles, revealing both the limitations of conventional antimicrobial therapies and the potential of host-directed strategies that restore organelle function and innate immunity.

## Therapeutic outlook

The increasing prevalence of drug-resistant mycobacterial diseases raises the urgent need for strategies that go beyond conventional antibiotics. HDTs that target mitochondria, ER, and Golgi apparatus may offer a promising alternative treatment option (Nasare and Bagade, 2025; Cumming et al, 2018) (Fig. 4).

*Mtb* and *M. abscessus* alter host mitochondrial metabolism, shifting it toward fatty acid-dependent respiration and enhanced OXPHOS, which has been associated with prolonged intracellular bacterial survival (Cumming et al, 2018; Kim et al, 2025; Medikonda et al, 2024). Excess TNF induces mitochondrial ROS *via* reverse electron transport, triggering necrosis in infected macrophages; this can be prevented by metformin, which restores mitochondrial respiration and enhances bacterial clearance (Roca et al, 2022; Tetteh et al, 2025; Patrick and Watson, 2021). Modulating mitochondrial dynamics further affects infection outcomes: DRP1 inhibitors or MFN2 activators stabilize organelles networks, maintain ATP generation, and promote apoptosis in infected cells (Kim et al, 2025; de Souza and Cavalcante 2022), while mitophagy activators (resveratrol and berberine compounds) remove damaged mitochondria and limit pathogen growth (Gao et al, 2025) (Fig. 4A). Mitochondria-targeted redox modulators such as Mito-NOX or MitoQ fine-tune mtROS, enhancing antimicrobial responses while protecting host cells (Poerio et al, 2022; Ellzey et al, 2023).

*Mtb* infection induces ER stress, as shown for the secreted effector CdhM, which perturbs ER morphology and activates the UPR, including BiP, CHOP, XBP1 splicing, and elF2α phosphorylation, and has been associated with increased macrophage apoptosis and enhanced bacterial replication (Xu et al, 2022). Pharmacological restoration of ER proteostasis using chemical chaperones, such as 4-phenylbutyrate (4-PBA) or tauroursodeoxycholic acid (TUDCA), has been shown to enhance macrophage viability and reduce intracellular mycobacterial loads by mitigating ER stress-mediated apoptosis (Almanza et al, 2019) (Fig. 4B). Sigma-1 receptor agonists, such as PRE-084, modulate calcium-dependent complex I activity at ER–mitochondria contacts and increase mtROS (Goguadze et al, 2019) (Fig. 4B).

Mycobacteria also affect the Golgi complex, where Rab6B, a Golgi-localized GTPase, regulates TNF-α secretion in macrophages. In *M. bovis* BCG-infected cells, Rab6-dependent trafficking is disrupted, which has been shown to reduce pro-inflammatory cytokine release and alter host immune responses (Domínguez Cadena et al, 2021). Strategies that preserve Golgi integrity or modulate Rab GTPase function restore cytokine trafficking and support antigen presentation, highlighting the Golgi as a critical node in host-pathogen interaction (Pal et al, 2022).

HDTs that enhance inter-organelle communication are emerging as promising strategies against mycobacterial infections. AMPK activation, as induced by troglitazone, promotes autophagy and enhances antimicrobial activity in macrophages *via* the LKB1–AMPK pathway, improving clearance of *Mtb* (Bi et al, 2025). Similarly, targeting mitochondrial metabolism, through modulation of fatty acid or glutamine oxidation, can restrict intracellular growth of *M. abscessus* and *Mtb* by restoring energy flux and ROS signaling (Kim et al, 2025; Patrick and Watson, 2021; Pellegrino et al, 2023). mTORC1 is a central metabolic regulator that suppresses autophagy under nutrient-replete conditions; its inhibition releases ULK1 and initiates autophagic flux, thereby enhancing intracellular antimicrobial pathways. In TB animal and cellular models, pharmacological activation of AMPK and consequent inhibition of mTORC1 (e.g., by metformin) promotes autophagy and improves control of *Mtb*, supporting mTOR modulation as a host-directed therapeutic strategy (Singhal et al, 2014; Deretic et al, 2013) (Fig. 4C). Lipid-based interventions, including PI5P-enriched liposomes, improve phagosomal acidification and ROS production, thereby enhancing bacterial killing in macrophages (Poerio et al, 2022). Host kinases, such as PRKCB, regulate phagosome-lysosome fusion, while microRNAs (anti-miRNA-27a, anti-miRNA-146a) modulate mitochondrial apoptosis and ER stress responses to counter pathogen-mediated immune suppression (Doghish et al, 2025; Zheng et al, 2025). Co-delivery of antibiotics with organelle-targeting nanoparticles further improves intracellular drug efficacy (Malik et al, 2024).

Human organoid and microfluidic granuloma models reveal that restoration of mitochondrial metabolism and ER–Golgi trafficking enhances epithelial and macrophage defenses against different species of mycobacteria (Leon-Icaza et al, 2023, 2025; Cahill et al, 2020; Domínguez Cadena et al, 2021). Complementary HDTs, including lipid metabolism modulators (ACC2/DGAT1 inhibitors, statins), vitamin D analogs, phosphodiesterase inhibitors, histone deacetylase inhibitors, and HIF-1α modulators, synergize with organelle-targeted strategies to repurpose host energy, promote autophagy, and restore antimicrobial gene expression (van der Klugt et al, 2025; Thomas et al, 2024).

Future HDTs for TB and NTM diseases are increasingly focused on orchestrating organelle synergy to restore host immune function (Leon-Icaza et al, 2025; Cahill et al, 2020). By reestablishing mitochondrial, ER, and Golgi signaling and metabolism, HDTs can transform infected macrophages from passive hosts into active defenders. Unlike conventional antibiotics, these approaches have the potential to shorten treatment duration, reduce toxicity, and minimize relapse (Silwal et al, 2021).

## Conclusion

Mycobacterial infection reveals a sophisticated battle over host organelle networks. Pathogenic mycobacteria such as *Mtb* alter mitochondrial, ER, and Golgi function, prompting intracellular changes associated with bacterial persistence, while the host engages integrated organelle crosstalk to coordinate metabolism, proteostasis, and immune signaling. This interplay underscores that effective therapy requires restoring cellular balance, not merely eliminating the pathogen. Host-directed interventions, targeting mitochondrial dynamics, ER stress responses, and lipid signaling, can reinforce organelle resilience, enhance macrophage bactericidal activity, and accelerate clearance. However, major questions remain regarding the precise mechanisms by which mycobacteria manipulate organelle networks and how these processes can be therapeutically exploited (see Box 1). For instance, the selective targeting of inter-organelle interactions, defining stress thresholds that determine macrophage fate, understanding metabolic reprogramming that suppresses immune activation, and identifying molecular regulators at the mitochondria-ER–Golgi interface all represent critical gaps. Addressing these questions will be essential to develop next-generation host-directed therapies that restore organelle communication, reverse pathogen-induced tolerance, and reinstate robust antimicrobial immunity. Combined with conventional antibiotics, such strategies offer a route to faster, relapse-free treatment. Beyond TB, this framework highlights a broader principle: harnessing and restoring the structural and functional integrity of host cells provides a versatile blueprint for combating diverse intracellular pathogens by strengthening the cell’s own defenses.

Box 1. **In need of answers** (i) Can specific inter-organelle interactions be selectively targeted to develop novel HDTs against mycobacteria?

(ii) What are the critical stress thresholds within mitochondria, the ER, or the Golgi that determine whether infected macrophages undergo apoptosis, autophagy, or remain viable pathogen reservoirs?

(iii) How does mycobacteria-driven metabolic reprogramming integrate with organelle signaling to suppress immune activation while preserving host cell viability?

(iv) By what mechanisms do mycobacteria reshape the coordinated mitochondria–ER–Golgi network to establish and maintain long-term intracellular persistence?

(v) How do disruptions in organelle crosstalk contribute to differential immune responses among macrophage subtypes, and can these pathways be modulated therapeutically?

(vi) To what extent can restoring organelle communication reverse pathogen-induced tolerance and reinstate effective antimicrobial immunity?

(vii) Are there undiscovered molecular regulators at the interface of these organelles that could serve as precision targets for next-generation HDTs?

## Supplementary information

Peer Review File
