# Supplementary material for: Organelle interplay in host responses and mycobacterial resilience
Source: EMBO Rep. 2026 Jun 18;27(14):3912–27. doi: 10.1038/s44319-026-00836-y (PMC13400743; doi:10.1038/s44319-026-00836-y)
Supplement: Supplementary file 1 — Peer Review File [file 44319_2026_836_MOESM1_ESM.pdf]

EMBO reports

Organelle interplay in host responses and mycobacterial resilience

Wassim Daher, Shriya SINGH, Valentin BERDAL, Chloé DEFLANDRE, Zhuoyan CHEN, and Laurent Kremer

Corresponding author(s): Wassim Daher (wassim.daher@irim.cnrs.fr)

Review Timeline:

Submission Date:	7th Jan 26
Editorial Decision:	23rd Feb 26
Revision Received:	30th Mar 26
Accepted:	5th Jun 26

Editor: Achim Breiling

Transaction Report:

(Note: With the exception of the correction of typographical or spelling errors that could be a source of ambiguity, letters and reports are not edited. Depending on transfer agreements, referee reports obtained elsewhere may or may not be included in this compilation. Referee reports are anonymous unless the Referee chooses to sign their reports.)

Dear Dr. Daher,

Thank you for the submission of your review article to our editorial offices. I have now received the full set of referee reports that is copied below. As you will see, two referees find that the review is nice and comprehensive and have only minor suggestions to improve the article. In contrast, referee #1 is rather critical, indicating also that the text contains mistakes. The report suggests that a major and careful revision of the manuscript would be necessary to allow publication of the review in EMBO reports.

Given these constructive referee comments, I would thus like to invite you to revise your manuscript with the understanding that all referee points will be addressed in the revised manuscript and in a detailed point-by-point response. Acceptance of your manuscript will depend on a positive outcome of a second round of review.

Please pay particular attention to the references, as indicated by referee #1. Please cite primary research as much as possible, avoid citations to reviews and opinion articles, and make sure that the cited references fit and state what is mentioned in the text.

Revised manuscripts should be submitted within three months of a request for revision. Please contact me to discuss the revision (also by video chat) if you have questions or comments regarding the revision, or should you need additional time.

I further have these editorial requests:

- Please restrict the number of keywords to 5 and move these below the abstract.

- We usually ask our authors to include a box (Box 1) called "In need of answers" that briefly outlines the major questions that are still open in a given field in the form of a few bullet points. These questions can be accompanied by a brief explanation of what would be needed to address them and may provide helpful towards setting the stage for future experimentation in the field. For an example see this recent review we published: https://www.embopress.org/doi/full/10.1038/s44319-024-00135-4

- Please also add callouts for the box to the manuscript text (Box 1).

- We now use CRediT to specify the contributions of each author in the journal submission system. CRediT replaces the author contribution section. Please use the free text box to provide more detailed descriptions and do NOT provide your final manuscript text file with an author contributions section. See also our guide to authors: https://www.embopress.org/page/journal/14693178/authorguide#authorshipguidelines

- Please also make sure the references and their callouts are formatted according to our reference format (with et al. for manuscripts with more than 10 authors): http://www.embopress.org/page/journal/14693178/authorguide#referencesformat

- Please make sure that all the funding information is also entered into the online submission system and that it is complete and similar to the one in the acknowledgement section of the manuscript text file.

- Please remove the Data Availability section. As this is a review, no large primary datasets have been deposited at external repositories.

I think this is a very interesting review and while I appreciate that incorporating the referees' suggestions will still require some work, I am convinced that the article is worth it and will benefit from it.

When submitting your revised manuscript, we will require a Microsoft Word file (.doc) of the revised manuscript text including detailed figure legends (at the very end), but without the figures.

Please provide the final figures as separate, high-resolution files as .pdf, .eps, .tif, or .jpg (one file per figure). Please finalize the drafts provided and make sure they accurately illustrate the key scientific concepts that you wish to show.

Please also note the following points:

- If there are certain aspects of your figure draft that are based upon assumptions or where the scientific data remains ambiguous (for example, schematically depicting a presumed direct protein-protein interaction, protein shape or subcellular localizations etc.) please add a comment so that we can work with you on an accurate depiction. Please ensure the directionality and nature of interactions is presented accurately.

- If the figure or single panels of the figure have been adapted from a published figure, please add this information to the figure legend (e.g., 'Adapted from...' or 'Based on...'). The editor will discuss if a reference and permission will be necessary

- Please only re-use figures or parts of a figure if this is essential for understanding the concept communicated. Often a reference to a previous paper will suffice. If the figure contains re-used images or elements of images, including schematics, micrographs or photos, please make sure that you have the permission/license to publish it (this also applies to your own previous work, if the journal you published in retains copyright. Certain 'creative commons' open access licenses, such as CC-BY 4.0, allow re-use without additional formal permissions). All re-used material must be explicitly cited.

- If you use an image data base for scientific iconography (e.g., BioRender), please let us know if you have a license that allows for publication in an academic journal. Often authors use misleading iconography for expedience. Please ensure the information shown is scientifically accurate. If in doubt, please discuss with the editor or provide a sketch so that our designers can create accurate iconography.

- For figures created using a software for editing vector objects like Inkscape, CorelDraw etc., please send the file as a PDF (or SVG, or EPS), PowerPoint or Keynote in which the labels and objects are still editable. For figures created using Adobe Illustrator, please send the Illustrator (.ai) file.

I look forward to seeing a revised version of your manuscript when it is ready. Please let me know if you have questions or comments regarding the revision.

Yours sincerely,

Achim Breiling
Senior Editor
EMBO Reports

Referee #1:

The manuscript by Singh et al. discusses the role of mitochondria, ER and Golgi during mycobacterial infections. The subject is interesting, but the manuscript has several shortcomings in its current form and contains serious mistakes.

The introduction is too simplistic by generalizing across mycobacterial species that express different virulence factors (e.g. type VII secretion systems, where e.g. Mtb expresses ESX-1 which can permeabilize host membranes including the phagosome and facilitate Mtb escape/access to the cytosol whereas M. avium, which does not express ESX-1, is confined to phagosomes). Moreover, granuloma formation cannot be attributed solely to inhibition of lysosomal fusion; rather, defective pathogen clearance in permissive cell types (e.g., AMs and PMNs), coupled with inflammation and host cell death, promotes the recruitment of innate and adaptive immune cells and granuloma development. This is better described in reviews by Chandra et al, https://doi.org/10.1038/s41579-022-00763-4, Warner et al https://doi.org/10.1038/s41579-025-01201-x and more. Similarly - the notion that mitochondria, ER and Golgi orchestrate innate and adaptive immunity is an exaggeration. Though these organelles are important players, the endo-lysosomal network and the cytosol are major compartments for pathogen sensing and signaling - some of which is mediated via docking/assembly of signaling components onto internal membranes (including the ER, mito and Golgi).

The manuscript contains several sections of repetitive text. Example (one of many) from page 8: "While mitochondria orchestrate many of the host's protective responses, mycobacterial pathogens, particularly Mtb, have adapted to exploit these very systems. Instead of merely resisting mitochondrial defenses, they actively reprogram mitochondrial metabolism, dynamics and signaling to sustain intracellular survival and chronic infection. Mitochondria are central to host defense, yet mycobacterial species have evolved sophisticated strategies to neutralize and exploit them for persistence and survival."

The lack of primary literature citations supporting claims made by the authors is a major problem. Most of the literature cited are reviews by others, which makes it difficult to verify conclusions drawn - especially since many primary studies show opposite results regarding pathogen- or host beneficial outcomes, e.g. cGAS/STING activation and type I IFN responses (and many more).

In some cases where primary literature is cited, the context or interpretation of the outcomes gets wrong. Ex from Page 9, which discusses Mtb strategies in manipulating mitochondria: Mtb also inhibits mitophagy via the PINK1-Parkin pathway, preventing clearance of depolarized mitochondria, reducing mtROS, and sustaining ATP production without triggering cell death (Gao et al, 2025; Ning et al, 2021). The Ning et al. study shows that Mtb-infection induces mitochondrial fusion, resulting in increased ATP and anti-microbial autophagy - and silencing of this process (siMFN1) severely reduced the antimicrobial capacity of the infected macrophages. There is no mentioning that Mtb manipulates this host-defensive mechanism to its advantage. Right after, lowering of ATP production is mentioned as an Mtb-induced beneficial effect.

Another example from page 10: "Altered calcium flux at ER-mitochondria contact sites weakens inflammasome activation, while Golgi fragmentation impairs cytokine secretion, a process that depends on adequate mitochondrial ATP supply (Domínguez

Cadena et al, 2021). The cited paper shows that Rab6b localizes to the Golgi complex in murine macrophages and promotes TNF release in response to M. bovis BCG infection - which is completely different from what the authors claim? The same study is cited again on pages 14-15 while claiming that Mtb disrupts the Golgi, impair vesicle formation and trafficking? The paper has 3 figures, all supporting Rab6b in promoting TNF production - and all with M. bovis BCG infection (not Mtb). This is seriously wrong.

On page 11 about ER stress, the authors cite studies on mycobacterial factors that induce ER stress - but contrary to the authors conclusions that Mtb targets the ER to promote survival, the papers show that ER stress is followed by apoptosis or autophagy, which are both antimicrobial? The study by Yu et al shows that PE_PGRS1 reduces ER stress and thereby increases Mtb survival. In the same section, the authors first say Mtb PE_PGRS1 activates the UPR and pro-inflammatory responses ... while a sentence later they say Mtb exploits the UPR to evade pro-inflammatory responses?

In general, authors should avoid attributing intention to bacteria, which is done throughout the manuscript. While host processed change upon Mtb-infection, these have developed over centuries of co-evolution and are adaptations to increase survival. In cases where Mtb virulence factors have been identified that perturb specific host responses, these can be described as such - but without purpose (ex - PE/PGRS proteins ... increase mitochondrial membrane permeability - to delay apoptosis, page 9, should be rewritten to "resulting in", or " was shown to delay" if the study shows so).

Referee #2:

The authors wrote a nice review an a topic that is underexplored in the host-pathogen interaction regarding mycobacteria and macrophages.
I have one major concern and that is the overuse of review articles as a reference. In my opinion, the heavy use of review articles is justified in the Introduction section and in all of the sections giving general cell biology background. All the paragraphs discussing mycobcteria related data should include ALL relevant primary literature citations. Otherwise, the article will be of less interest to most readers.

Referee #3:

The review "Organelle interplay in host responses and mycobacterial resilience" provides discussion highlighting the ways in which organelle functions and crosstalk become dysregulated or reappropriated during mycobacterial infection, an important topic that underscores the complexity of mycobacterial pathogenesis as it occurs in vivo. The authors introduce the field and review some of its major topics, including the roles of mitochondria, endoplasmic reticulum, the Golgi apparatus, inter-organelle cross talk, and potential therapeutic related to these systems. I did not fully appreciate the scale of organelle remodeling, functional roles, and cross-talk, and I believe this work will make a valuable contribution to understanding not only mycobacterial pathogenesis but also the biology of intracellular pathogens more broadly. I particularly appreciated the final figure and the discussion of the role of HDTs. Overall, the review is well-written and expertly informed, but may benefit from minor edits to improve clarity and readability. The review is written in a detailed but digestible manner suitable for a general scientific audience, and will be an important resource for the mycobacterial research community.

Major comments and questions
• Significant work has focused on the role of nitric oxide (NO) in controlling mycobacterial infection, but there is no discussion of NO in this review. Would Because of the role of NO in stimulating host immune responses, some mention may be relevant.
• The authors also discuss the importance of reactive oxygen species (ROS) in signaling and in bactericidal activity. There is a body of work discussing the importance of metal homeostasis in mycobacterial infection. Given the relationship between biologically important transition metals and redox homeostasis, is there a known role for metal flux in influencing the role of ROS?
• Section v.2 felt particularly lacking in detail- i.e., what other intracellular pathogens subvert golgi trafficking?

Minor comments and questions
• In the intro, the statistics on cases and deaths may be outdated and should reference the WHO annual report
• Parts of the manuscript felt repetitive.

Editorial requests:

- Please restrict the number of keywords to 5 and move these below the abstract.

We have restricted the number of keywords to five and placed them below the abstract as requested. The revised keywords are: Mycobacterium, Host–pathogen interactions, Mitochondria, Host organelles, Immune–oxidative responses.

- We usually ask our authors to include a box (Box 1) called "In need of answers" that briefly outlines the major questions that are still open in a given field in the form of a few bullet points. These questions can be accompanied by a brief explanation of what would be needed to address them and may provide helpful towards setting the stage for future experimentation in the field. For an example see this recent review we published: https://www.embopress.org/doi/full/10.1038/s44319-024-00135-4

We have added Box 1: "In need of answers" to the manuscript, listing the major open questions in the field as bullet points.

- Please also add callouts for the box to the manuscript text (Box 1).

Done. A callout to Box 1 has been added in the conclusion to highlight the remaining open questions and connect them to the discussion of host organelle interactions and therapeutic opportunities.

- We now use CRediT to specify the contributions of each author in the journal submission system. CRediT replaces the author contribution section. Please use the free text box to provide more detailed descriptions and do NOT provide your final manuscript text file with an author contributions section. See also our guide to authors:
https://www.embopress.org/page/journal/14693178/authorguide#authorshipguidelines

Done. We have updated the submission to use CRediT taxonomy in the journal system, providing detailed author contribution descriptions in the free text box. The manuscript no longer includes a separate author contributions section.

- Please also make sure the references and their callouts are formatted according to our reference format (with et al. for manuscripts with more than 10 authors):
http://www.embopress.org/page/journal/14693178/authorguide#referencesformat

We have reviewed all references in the manuscript and formatted them according to the journal's guidelines, using "et al." for manuscripts with more than 10 authors.

- Please make sure that all the funding information is also entered into the online submission system and that it is complete and similar to the one in the acknowledgement section of the manuscript text file.

All funding information has been entered into the online submission system and has been checked to ensure it is complete and matches the acknowledgements section in the manuscript text.

- Please remove the Data Availability section. As this is a review, no large primary datasets have been deposited at external repositories.

Done. The Data Availability section has been removed, as the manuscript is a review and no primary datasets are deposited.

I think this is a very interesting review and while I appreciate that incorporating the referees' suggestions will still require some work, I am convinced that the article is worth it and will benefit from it.

Thank you for your positive feedback. We have carefully considered all referees' comments and modified the text accordingly. We believe these revisions have improved the manuscript and strengthened its overall clarity and impact.

When submitting your revised manuscript, we will require a Microsoft Word file (.doc) of the revised manuscript text including detailed figure legends (at the very end), but without the figures.
Thank you for the clarification. We have submitted the revised manuscript as a Microsoft Word file (.doc), including all detailed figure legends at the end, but without the figures, as requested.

Please provide the final figures as separate, high-resolution files as .pdf, .eps, .tif, or .jpg (one file per figure). Please finalize the drafts provided and make sure they accurately illustrate the key scientific concepts that you wish to show.
We have provided the final figures as separate, high-resolution PDF files, with one file per figure, accurately illustrating the key scientific concepts of the manuscript.

Please also note the following points: - If there are certain aspects of your figure draft that are based upon assumptions or where the scientific data remains ambiguous (for example, schematically depicting a presumed direct protein-protein interaction, protein shape or subcellular localizations etc.) please add a comment so that we can work with you on an accurate depiction. Please ensure the directionality and nature of interactions is presented accurately.
Please note that this point is not applicable to our figures, as all interactions, localizations, and features depicted are based on established scientific data rather than assumptions.

- If the figure or single panels of the figure have been adapted from a published figure, please add this information to the figure legend (e.g., 'Adapted from...' or 'Based on...'). The editor will discuss if a reference and permission will be necessary
Not applicable: none of our figures or panels have been adapted from previously published figures.

- Please only re-use figures or parts of a figure if this is essential for understanding the concept communicated. Often a reference to a previous paper will suffice. If the figure contains re-used images or elements of images, including schematics, micrographs or photos, please make sure that you have the permission/license to publish it (this also applies to your own previous work, if the journal you published in retains copyright. Certain 'creative commons' open access licenses, such as CC-BY 4.0, allow re-use without additional formal permissions). All re-used material must be explicitly cited.
Not applicable: all figures in our manuscript are original and have not been re-used from previous publications.

- If you use an image data base for scientific iconography (e.g., BioRender), please let us know if you have a license that allows for publication in an academic journal. Often authors use misleading iconography for expedience. Please ensure the information shown is scientifically accurate. If in doubt, please discuss with the editor or provide a sketch so that our designers can create accurate iconography.
We used BioRender for our figure schematics and confirm that we have a license allowing publication in an academic journal. All iconography has been checked for scientific accuracy.

- For figures created using a software for editing vector objects like Inkscape, CorelDraw etc., please send the file as a PDF (or SVG, or EPS), PowerPoint or Keynote in which the labels and objects are still editable. For figures created using Adobe Illustrator, please send the Illustrator (.ai) file.
Not applicable, our figures were created using BioRender, not vector-editing software.

Referee #1:

The manuscript by Singh et al. discusses the role of mitochondria, ER and Golgi during mycobacterial infections. The subject is interesting, but the manuscript has several shortcomings in its current form and contains serious mistakes.

The introduction is too simplistic by generalizing across mycobacterial species that express different virulence factors (e.g. type VII secretion systems, where e.g. Mtb expresses ESX-1 which can permeabilize host membranes including the phagosome and facilitate Mtb escape/access to the cytosol whereas M. avium, which does not express ESX-1, is confined to phagosomes). Moreover, granuloma formation cannot be attributed solely to inhibition of lysosomal fusion; rather, defective pathogen clearance in permissive cell types (e.g., AMs and PMNs), coupled with inflammation and host cell death, promotes the recruitment of innate and adaptive immune cells and granuloma development. This is better described in reviews by Chandra et al, https://doi.org/10.1038/s41579-022-00763-4, Warner et al https://doi.org/10.1038/s41579-025-01201-x and more.

We thank the reviewer for this important point. We have revised the introduction to clarify that virulence strategies differ between mycobacterial species. In particular, *M. tuberculosis* expresses ESX-1, which can mediate contact-dependent phagosomal and host membrane disruption, whereas *M. avium* lacks ESX-1 and is largely restricted to phagosomal compartments that mature to late endosomes/phagolysosomes (Conrad et al, 2017; Gidon et al, 2017). We have also clarified that granuloma formation results from a combination of defective pathogen clearance, inflammation, and host cell death, rather than being solely attributable to phagosome-lysosome fusion inhibition (Chandra et al, 2022; Warner et al, 2025). These changes address the reviewer's concern regarding oversimplification and generalization across species.

Similarly - the notion that mitochondria, ER and Golgi orchestrate innate and adaptive immunity is an exaggeration. Though these organelles are important players, the endo-lysosomal network and the cytosol are major compartments for pathogen sensing and signaling, some of which is mediated via docking/assembly of signaling components onto internal membranes (including the ER, mito and Golgi).

We thank the reviewer for this valuable comment. We have revised the text to clarify that the endo-lysosomal network and cytosol are the primary compartments for pathogen sensing and signaling, and that signaling often occurs via docking and assembly of receptors and downstream molecules on internal membranes, including the ER, mitochondria, and Golgi (Nakamura et al, 2014; Miyake et al, 2019). While mitochondria, ER, and Golgi contribute to immune signaling, their roles are integrated with endo-lysosomal and cytosolic pathways rather than acting as sole orchestrators. These changes address the reviewer's concern about oversimplification and better reflect current understanding of host-pathogen interactions.

The manuscript contains several sections of repetitive text. Example (one of many) from page 8: "While mitochondria orchestrate many of the host's protective responses, mycobacterial pathogens, particularly Mtb, have adapted to exploit these very systems. Instead of merely resisting mitochondrial defenses, they actively reprogram mitochondrial metabolism, dynamics and signaling to sustain intracellular survival and chronic infection. Mitochondria are central to host defense, yet mycobacterial species have evolved sophisticated strategies to neutralize and exploit them for persistence and survival."

We thank the reviewer for pointing out the presence of repetitive text. We have carefully reviewed the manuscript and revised the sections to remove redundancy wherever possible. Specifically, passages describing the interplay between mitochondria and mycobacterial manipulation have been consolidated and rewritten to maintain clarity while avoiding repetition. The text now emphasizes mechanistic insights and therapeutic implications without reiterating the same points multiple times. Overall, the text has been checked and modified throughout the review to ensure a concise and streamlined narrative, improving readability and maintaining focus on key conceptual advances.

The lack of primary literature citations supporting claims made by the authors is a major problem. Most of the literature cited are reviews by others, which makes it difficult to verify conclusions drawn - especially since many primary studies show opposite results regarding pathogen- or host beneficial outcomes, e.g. cGAS/STING activation and type I IFN responses (and many more).
We thank the reviewer for highlighting the reliance on review articles. We have revised the manuscript to extensively cite primary literature for all mechanistic claims, including, for example, cGAS/STING-mediated type I interferon responses. The revised text now incorporates studies demonstrating both protective and non-essential roles of cGAS/STING in *M. tuberculosis* and nontuberculous mycobacterial infections (Watson et al., 2015; Wassermann et al., 2015; Ding et al., 2024; Ruangkiattikul et al., 2017; Marinho et al., 2018). The paragraph has been restructured to accurately reflect these context-dependent outcomes, and the manuscript was carefully checked and modified to remove repetitive statements. Where possible, we added numerous new primary research articles in addition to reviews. We believe these changes substantially improve the accuracy, verifiability, and clarity of the manuscript.

In some cases where primary literature is cited, the context or interpretation of the outcomes gets wrong. Ex from Page 9, which discusses Mtb strategies in manipulating mitochondria: Mtb also inhibits mitophagy via the PINK1-Parkin pathway, preventing clearance of depolarized mitochondria, reducing mtROS, and sustaining ATP production without triggering cell death (Gao et al, 2025; Ning et al, 2021). The Ning et al. study shows that Mtb-infection induces mitochondrial fusion, resulting in increased ATP and anti-microbial autophagy - and silencing of this process (siMFN1) severely reduced the antimicrobial capacity of the infected macrophages. There is no mentioning that Mtb manipulates this host-defensive mechanism to its advantage. Right after, lowering of ATP production is mentioned as an Mtb-induced beneficial effect.
We thank the reviewer for pointing out the misinterpretation regarding *Mtb* manipulation of mitophagy and mitochondrial metabolism. The paragraph on apoptosis inhibition and maintenance of the replicative niche has been substantially revised. In particular, the previously cited Ning et al. study has been removed, as it describes a host-protective mechanism rather than bacterial exploitation, and its interpretation in the original text was inaccurate. The Gao et al. study, which provides relevant mechanistic insight into mitophagy during *Mtb* infection, has been retained. The revised paragraph now clearly distinguishes host-defense processes from bacterial strategies, avoids misinterpretation of primary data, and accurately reflects the current understanding of mitophagy, ATP maintenance, and *Mtb*-mediated modulation of apoptosis and necroptosis. These changes ensure that all mechanistic claims are properly supported by primary literature and that the text accurately conveys context-dependent outcomes.

Another example from page 10: "Altered calcium flux at ER-mitochondria contact sites weakens inflammasome activation, while Golgi fragmentation impairs cytokine secretion, a process that depends on adequate mitochondrial ATP supply (Domínguez Cadena et al, 2021). The cited paper shows that Rab6b localizes to the Golgi complex in murine macrophages and promotes TNF release in response to M. bovis BCG infection - which is completely different from what the authors claim? The same study is cited again on pages 14-15 while claiming that Mtb disrupts the Golgi, impair vesicle formation and trafficking? The paper has 3 figures, all supporting Rab6b in promoting TNF production - and all with M. bovis BCG infection (not Mtb). This is seriously wrong.
We thank the reviewer for identifying the misattribution regarding mitochondrial–Golgi crosstalk and cytokine secretion. Upon careful review, we recognized that the Domínguez Cadena et al. study reports Rab6b-mediated TNF release in murine macrophages infected with *M. bovis* BCG and does not support the claims about Golgi fragmentation or Mtb-induced impairment of vesicle trafficking. Accordingly, we have removed this reference from the paragraph and revised the text and figures to accurately reflect the

primary literature. The modified paragraph now cites studies that directly support Mtb-induced perturbation of mitochondrial dynamics, ER-mitochondria contact sites, calcium flux, inflammasome activation, redox metabolism, and mitochondrial quality control, without overstating the role of the Golgi or relying on unrelated BCG studies. These revisions ensure that all mechanistic claims are consistent with the cited primary literature and that the text accurately reflects current understanding of Mtb-mediated mitochondrial subversion. We believe these changes address the reviewer's concerns and strengthen both the accuracy and verifiability of the manuscript.

On page 11 about ER stress, the authors cite studies on mycobacterial factors that induce ER stress - but contrary to the authors conclusions that Mtb targets the ER to promote survival, the papers show that ER stress is followed by apoptosis or autophagy, which are both antimicrobial? The study by Yu et al shows that PE_PGRS1 reduces ER stress and thereby increases Mtb survival. In the same section, the authors first say Mtb PE_PGRS1 activates the UPR and pro-inflammatory responses ... while a sentence later they say Mtb exploits the UPR to evade pro-inflammatory responses?
We thank the reviewer for pointing out the inconsistencies regarding ER stress and UPR-driven responses. In response, we carefully reviewed the content of each cited study, corrected misinterpretations, and revised the paragraph accordingly. References that did not accurately support the claims were removed, and new primary literature was added to ensure all statements faithfully reflect experimental findings. The revised paragraph now clearly distinguishes between mycobacterial effectors that activate ER stress leading to host-protective responses (e.g., apoptosis or autophagy) and effectors that modulate or dampen UPR signaling to promote intracellular survival. For instance, PE_PGRS1 is correctly described as reducing ER stress and limiting pro-apoptotic signaling, thereby enhancing bacterial survival, while other effectors such as Rv0297 can trigger UPR-driven apoptosis that may facilitate dissemination rather than clearance. This restructuring resolves the previous contradiction and emphasizes that *Mtb* exploits UPR plasticity in a context-dependent manner, rather than uniformly activating ER stress for survival. Overall, these modifications improve accuracy, align conclusions with primary data, and provide a clearer mechanistic framework for how *Mtb* interacts with host ER stress pathways.

In general, authors should avoid attributing intention to bacteria, which is done throughout the manuscript. While host processed change upon Mtb-infection, these have developed over centuries of co-evolution and are adaptations to increase survival. In cases where Mtb virulence factors have been identified that perturb specific host responses, these can be described as such - but without purpose (ex - PE/PGRS proteins ... increase mitochondrial membrane permeability - to delay apoptosis, page 9, should be rewritten to "resulting in", or " was shown to delay" if the study shows so).
We thank the reviewer for highlighting the concern regarding attributing intentionality to *M. tuberculosis*. We agree that host-pathogen interactions should be described based on observed outcomes rather than implied bacterial purpose. In response, we have revised the manuscript throughout to remove language suggesting bacterial "intent" or "strategy." These changes were applied systematically to all sections discussing virulence factors and host responses, ensuring that the manuscript accurately describes mechanistic effects without implying purposeful action by the bacteria.

Referee #2:
The authors wrote a nice review on a topic that is underexplored in the host-pathogen interaction regarding mycobacteria and macrophages. I have one major concern and that is the overuse of review articles as a reference. In my opinion, the heavy use of review articles is justified in the Introduction section and in all of the sections giving general cell biology background. All the paragraphs discussing mycobcteria related data should include ALL relevant primary literature citations. Otherwise, the article will be of less interest to most readers.

We thank the reviewer for the positive evaluation of our review and for highlighting the concern regarding the overuse of review articles. We agree that primary literature should support mechanistic claims, particularly in sections discussing mycobacteria-specific host-pathogen interactions. In response, we have carefully revised the manuscript: all paragraphs addressing *M. tuberculosis*-related experimental data now include extensive citations to primary literature, while review articles are retained primarily in the Introduction and sections providing general cell biology background. Where necessary, references were updated, and statements were cross-checked to ensure they accurately reflect the findings of the cited primary studies. We believe these revisions improve the verifiability and value of the review, making it more informative and useful for readers interested in host-pathogen interactions at a mechanistic level.

Referee #3:

The review "Organelle interplay in host responses and mycobacterial resilience" provides discussion highlighting the ways in which organelle functions and crosstalk become dysregulated or reappropriated during mycobacterial infection, an important topic that underscores the complexity of mycobacterial pathogenesis as it occurs in vivo. The authors introduce the field and review some of its major topics, including the roles of mitochondria, endoplasmic reticulum, the Golgi apparatus, inter-organelle cross talk, and potential therapeutic related to these systems. I did not fully appreciate the scale of organelle remodeling, functional roles, and cross-talk, and I believe this work will make a valuable contribution to understanding not only mycobacterial pathogenesis but also the biology of intracellular pathogens more broadly. I particularly appreciated the final figure and the discussion of the role of HDTs. Overall, the review is well-written and expertly informed, but may benefit from minor edits to improve clarity and readability. The review is written in a detailed but digestible manner suitable for a general scientific audience, and will be an important resource for the mycobacterial research community.

We sincerely thank the reviewer for the positive and constructive evaluation of our review. We are pleased that the discussion of organelle remodeling, inter-organelle crosstalk, and host-directed therapies was appreciated, and that the review was considered clear, detailed, and accessible to a general scientific audience. We have carefully considered the suggestion regarding minor edits to improve clarity and readability and have revised the manuscript accordingly, streamlining sections where appropriate and ensuring consistent terminology and flow throughout the text.

Major comments and questions:

• Significant work has focused on the role of nitric oxide (NO) in controlling mycobacterial infection, but there is no discussion of NO in this review. Would Because of the role of NO in stimulating host immune responses, some mention may be relevant.

We agree that nitric oxide (NO) plays an important role in the immune response to *M. tuberculosis*. We have now added new sentences discussing the roles of NO and inducible nitric oxide synthase (iNOS) in the revised manuscript. Studies show that NO contributes to macrophage control of *M. tuberculosis*, modulates host signaling (e.g., via HIF-1α and NF-κB), and influences immunopathology by restraining inflammasome activation and neutrophil-driven inflammation. Relevant citations have also been included.

• The authors also discuss the importance of reactive oxygen species (ROS) in signaling and in bactericidal activity. There is a body of work discussing the importance of metal homeostasis in mycobacterial infection. Given the relationship between biologically important transition metals and redox homeostasis, is there a known role for metal flux in influencing the role of ROS?

We thank the reviewer for this insightful comment. We have revised the manuscript to include a new paragraph describing the relationship between metal flux and ROS in *Mycobacterium tuberculosis* infection. The new paragraph highlights how host-mediated metal manipulation influences ROS generation and bactericidal activity, and how mycobacteria employ metallostasis and redox-sensitive

regulatory mechanisms to counteract oxidative stress. Relevant studies, including those on iron, copper, and zinc homeostasis, as well as redox-sensitive factors such as WhiB3, have been cited to directly address the role of transition metals in modulating ROS during infection.

• Section v.2 felt particularly lacking in detail- i.e., what other intracellular pathogens subvert golgi trafficking?

We thank the reviewer for the comment. Section V.2 no longer exists in the revised manuscript. We have replaced it with a single paragraph in the section now entitled "V. INTER-ORGANELLE CROSS-TALK IN HOST DEFENSE AND MYCOBACTERIAL SUBVERSION", which focuses exclusively on literature related to *Mycobacterium* infection. Since the scope of our review is limited to mycobacterial infections, we chose not to extend this section to other intracellular pathogens that subvert Golgi trafficking. The revised section discusses how *Mycobacterium tuberculosis* perturbs mitochondria-ER contact sites (MERCs) and ER-Golgi trafficking, highlighting pathogen-induced modulation of host organelles, metabolic pathways, apoptosis, and immune signaling. We believe this focused approach better aligns with the aims of the review while maintaining mechanistic detail relevant to mycobacterial pathogenesis.

Minor comments and questions:

• In the intro, the statistics on cases and deaths may be outdated and should reference the WHO annual report.

We thank the reviewer for this important comment. The Introduction section of the manuscript has been revised to incorporate the most recent data from the *Global Tuberculosis Report 2025* published by the World Health Organization. Specifically, the epidemiological estimates have been updated to reflect that, in 2024, an estimated 10.7 million people (95% UI: 9.9–11.5 million) developed TB and 1.23 million (95% UI: 1.13–1.33 million) died from the disease worldwide. This updated information has now replaced the previously cited statistics in the revised manuscript.

• Parts of the manuscript felt repetitive.

We thank the reviewer for pointing this out. In response, we have carefully revised the manuscript to remove repetitive statements wherever possible, restructured paragraphs to improve flow, and consolidated overlapping content. Particular attention was given to sections discussing mitochondrial, ER, and Golgi functions, as well as host-directed therapies, to ensure that each concept is presented clearly and succinctly without unnecessary repetition.

Dr. Wassim Daher
Institut de Recherche en Infectiologie de Montpellier
CNRS, UMR9004
1919, route de Mende, UMR9004, 34293 Montpellier cedex 5 FRANCE
MONTPELLIER 34293
France

Dear Dr. Daher,

Thank you for the submission of your revised review manuscript to our editorial offices. I have now received the reports from the two referees that were asked to re-assess the manuscript, you will find below. As you will see, the referees now support the publication of the review in EMBO reports.

I am thus pleased to inform you that your manuscript has been accepted for publication in EMBO reports. Your manuscript will be processed for publication by EMBO Press. It will be copy edited and you will receive page proofs prior to publication. Please note that you will be contacted by Springer Nature Author Services to complete licensing information.

Should you be planning a Press Release on your article, please notify the production team via this form in order to coordinate publication and release dates: https://forms.cloud.microsoft/e/eH4A8JVP6g

If you have any questions, please do not hesitate to contact the Editorial Office. Thank you for your contribution to EMBO Reports.

Yours sincerely,

Achim Breiling
Senior Editor
EMBO Reports

Referee #2:

I think the revised version addresses my concerns in regard to the overuse of review articles. I support publication of the review.

Referee #3:

The authors have addressed the reviews well. I recommend publishing.

>>> Please note that it is EMBO Reports policy for the transcript of the editorial process (containing referee reports and your response letter) to be published as an online supplement to each paper. If you do NOT want this, you will need to inform the Editorial Office via email immediately. More information is available here: https://link.springer.com/partners/embo-press/editorial-policies#Peer%20review
